# Gastroenterology in the Metaverse: The dawn of a new era?

**DOI:** 10.3389/fmed.2022.904566

**Published:** 2022-08-11

**Authors:** Chi Zhang, Shuyan Feng, Ruonan He, Yi Fang, Shuo Zhang

**Affiliations:** ^1^The First Clinical Medical College, Zhejiang Chinese Medical University, Hangzhou, China; ^2^The Second Affiliated Hospital of Zhejiang Chinese Medical University, Hangzhou, China

**Keywords:** Metaverse, gastroenterology, artificial intelligence (AI), virtual reality, endoscope

## Abstract

2021 is known as the first Year of the Metaverse, and around the world, internet giants are eager to devote themselves to it. In this review, we will introduce the concept, current development, and application of the Metaverse and the use of the current basic technologies in the medical field, such as virtual reality and telemedicine. We also probe into the new model of gastroenterology in the future era of the Metaverse.

## Introduction

The term “Metaverse” became widely known in 2021, yet many individuals were unfamiliar with it. Almost everyone wants to know what the Metaverse is. The Metaverse is a difficult notion to grasp. Metaverse is a portmanteau of meta, meaning transcendent, and verse, from universe. The Metaverse is a collective virtual shared space that includes the collection of virtual worlds, expanding reality and the internet. It uses a variety of new generation information technologies to achieve the multidimensional and deep integration of technology, individual, organization, production, culture, social interaction, entertainment, and economy and constructs a new immersive network social form that is parallel and intermingled with human society and can be shared continuously (1, 2). In Neal Stephenson's science fiction novel Avalanche, published in 1992, the player takes on the role of a bespoke avatar, similar to the Matrix, Ready Player One's oasis, and Free Guy's free city. People in the Metaverse are not bound by the rules of nature and can break free from the restrictions of time and space. People can modify their roles in the Metaverse, such as age, gender, look, and even species, with the use of a range of virtual reality gadgets, open Second Life, and achieve all kinds of things that are impossible to attain in real life (3). The Metaverse concept has evolved, and it is now frequently coupled with new technologies such as extended reality (XR) (4), blockchain (5), cloud computing (6), and digital twinning (7). The hardware and software foundation of the Metaverse has matured with the development of 5th Generation Mobile Communication Technology (5G) (8), virtual reality (VR), augmented reality (AR), and artificial intelligence (AI) (9) as well as the significant improvements in computing power and graphics capability of computer chips. With a slew of major internet companies declaring their forays into the Metaverse, it is expected that the Metaverse will grow by leaps and bounds over the next decade.

Many disciplines will be transformed by the rise of the Metaverse. Although the Metaverse is presently mostly used in gaming, social media, and other industries, there is no doubt that when the Metaverse matures, it will have a significant impact on traditional industry. Medicine is a centuries-old profession with a long and illustrious history. The development of the Metaverse will fundamentally alter modern medicine as we know it. Whether it is the treatment process between doctors and patients, medical students' training method, or even the limitation of space, professionals from different countries and regions can engage in the same operation thanks to the Metaverse bridge between virtual and reality. At present, especially under the circumstance of the epidemic, many gastrointestinal endoscopists lack opportunities to go to developed countries for long-term professional training and learn the latest endoscopic technology due to the limitations of time and space (10). However, we can incarnate virtual characters in the Metaverse by using VR, AR, and other technologies. Instead of only observing the endoscopic screen, we could learn the expert's operation on the spot by watching a live broadcast of an endoscopy expert's operation on the other side of the world. On May 29, 2021, a Metaverse lung cancer training operation occurred in the intelligent classroom of Seoul National University in South Korea. A head-mounted display allowed up to 200 Asian thoracic doctors to access the Metaverse. Participants attended a virtual classroom after completing their customized settings and saw a 360-degree training session for lung cancer surgery and a presentation on metasomatic technology trends. Experts communicate with each other in real time through their avatars while watching surgical training and discuss procedures in the Metaverse while watching surgical training. Despite its tiny size, the metaverse is incredibly noteworthy, and this is the first report of its use in surgical training anywhere in the world (11). Furthermore, gastrointestinal endoscopists can use the vast internet database to look for the basic subject knowledge and training courses required in the Metaverse. They can even use sophisticated endoscopic equipment to gain access to the Metaverse and practice on virtual patients. Furthermore, the world still has a large number of less developed countries and regions with a severe shortage of digestive physicians and endoscopic equipment, and senior doctors can treat these patients *via* the internet and provide remote consultation, allowing high-quality medical resources to benefit more civilians and, to some extent, alleviating the problems associated with today's uneven distribution of medical resources. Of course, these concepts are not simply pipe dreams because the Metaverse's underlying technologies, such as virtual reality, tactile gloves, remote operation robots, and wireless communication technology, have advanced rapidly and have been employed in clinical diagnosis and treatment. By introducing the application of these basic techniques of Metaverse in gastroenterology, we could explore the prospect of Metaverse in gastroenterology in the future.

## Gastroenterology in the Metaverse

### The application of VR makes it possible for us to train endoscopic techniques in Metaverse

Virtual reality (VR), one of the meta-subtechnologies, has been used extensively in endoscopic physician training for at least 20 years (12). GI Mentor, the first generation of virtual endoscopy simulators, was used to train endoscopic trainee physicians as early as 2002 (13). When the endoscope is inserted into the GI model, the computer generates a three-dimensional view of the simulated digestive tract. At the same time, the force feedback module will mimic resistance anytime the simulated gastrointestinal wall is touched, providing a genuine experience during the surgery. The enhanced Pentax ECS-3840F allows users to perform the procedure, which enhances the endoscopic level of beginner physicians in a short period. Accutouch, a virtual reality colonoscopy simulation technology, was developed in 2005 to help professionals perform better in endoscopic colorectal cancer surgery (14). In comparison to GI Mentor, it offers clinicians a more realistic operation experience as well as a multimedia teaching and guidance system. Beginners can complete the colonoscopy training by following computer instructions. The Olympus Simulator for Colonoscopy (Endo TS1; The advent of Olympus KeyMed, Southend, UK) was released in 2008, allowing junior endoscopists to have a more realistic tactile experience during virtual reality endoscopy training (15). With the advancement of VR technology in recent years, an increasing number of powerful VR digestive endoscopy simulators have been used in clinical practice. In 2015, a new version of GI Mentor ii clinical simulation training was introduced (16). It may also replicate a wide range of colorectal illnesses, delivering a more realistic endoscopic experience. An increasing number of clinical studies have confirmed that virtual reality endoscopy training can significantly enrich doctors' experience, improve the level of endoscopic operation, and therefore shorten the time spent on it and decrease the incidence of surgical complications (17–20). Studies of the Erlangen Endo trainer have shown that simulator-based training has a positive effect on certain interventional skills, such as endoscopic haemostasis, perforation closure, and retrograde cholangiography (21). Of course, such models are not without flaws: to begin with, the VR model's diseases are limited, focusing primarily on routine examinations, endoscopic haemostasis, and closed perforation, such as easy operation training; nevertheless, it is difficult to replicate some challenging operations, such as endoscopic mucosal resection (EMR), endoscopic submucosal dissection (ESD), and endoscopic ultrasonography (EUS) (22). Furthermore, while such training is beneficial to beginning endoscopists, it does not raise the level of senior endoscopists (23, 24). As shown, the current generation of endoscopic simulators only offers basic training for endoscopists and is unable to replicate more complex surgical procedures, such as EMR, ESD or other treatment procedures (25), which significantly restricts the development of endoscopists. However, the Metaverse can eventually offer us all the scenarios we need to mimic, allowing us to overcome the constraints of models and technology because it is a virtual world that is entirely separate from the real world. Only endoscopists have access to Metaverse devices, can utilize VR helmets and tactile gloves, and have finished advanced endoscopic training. Additionally, we may use artificial intelligence (AI) to search for professional training videos in cloud databases and master the close-up endoscope treatment operation abilities in Metaverse (26). As a result, we can prevent the needless risk of surgical failure in addition to gaining extensive endoscopic expertise.

### The tactile glove helped us 'touch' the Metaverse

The user experience of the Metaverse will be significantly affected if users cannot have an experience that is close to reality (27); thus, the Metaverse is expected to give endoscopists a more realistic endoscopy experience, whether it be the damping feeling of the mirror body rotation or the feedback feeling of the adjustment knob that is highly crucial for an endoscopic treatment to ensure that endoscopy training is unique (28). META has introduced a glove for the Metaverse; when people put it on and enter the VR world, the complex control system on the tool adjusts inflation to create realistic pressure on their hands. Upon touching the virtual objects, one will feel certain tactile sensations, and when picking up a virtual object, the sensors on one's fingers become stiffened, creating a damping sensation. These sensations work in tandem with the visual and auditory interactions provided by the VR headset, creating the illusion of physical contact with an object (29). Interestingly, this type of haptic glove is not a recent invention. As early as 2003, scholars in basic neuroscience invented a tactile glove. The gloves can be combined with technologies such as functional magnetic resonance imaging (fMRI) to identify brain activities behind the complex behaviors and use their built-in vibration-stimulation device to provide precise tactile feedback to doctors (30). While the gloves at the time were insufficient to create fairly accurate tactics, they lifted the potential for physical rehabilitation by virtue of VR for people who were not able to move. The development of haptic gloves has made a huge breakthrough in recent years. A microfluidic diaphragm pressure sensor was first introduced in 2017. When touching or gripping objects, haptic gloves equipped with this sensor can give the hand a more comprehensive and realistic tactile sensation. In 2019, Indian scientists developed ultraviolet- and ray-resistant biologically inspired skin that can also be self-cleaning. It imitates the pressure-sensing capabilities of human skin, allowing it to record details and provide a mechanical sensation when in use. The tactile glove will dramatically improve the authenticity of the tactile sense by using biologically inspired skin, allowing users to have a more genuine experience in the metaversity (31). A research team presented a remote palpation device based on a haptic glove gadget the same year. A remote surgeon can remotely palpate the softness or hardness of nodules or tissues using a remote surgical platform and a haptic glove. The accuracy of the remote platform has been tested to be approximately 74% (32). A researcher devised and proposed a low-delay haptic open glove (LLHOG) based on a rotating position sensor and a minimum-maximum zoom (MMS) filter in 2021 and utilized it to provide immersive virtual reality engagement. The open glove detects finger flexion and adduction movements using two position sensors at the metacarpophalangeal joint (MCP), with a processing delay of 145.37 microseconds and an overall hand motion tracking delay of 4 milliseconds (33). Of course, tactile gloves are inextricably linked to the virtual reality interactive simulation system, no matter how powerful they are (34). As an important medium for people to experience the Metaverse, the continuous development of tactile gloves can not only provide a more realistic touch for everyone who experiences the metaverse, but more importantly, it will lay a solid foundation for the future application of the Metaverse in the field of gastroenterology (35). It can be said that only when haptic gloves achieve more accurate feedback and lower latency can virtual endoscopic training and even future remote endoscopic surgery truly achieve truly ideal results. There are still considerable problems with the current tactile gloves. The most fatal factor is the limitation of the number of receptors. To achieve near-real touch, each glove needs at least thousands of brakes. However, the current gloves are limited by weight and cost; too many sensors will greatly increase the weight and cost of the glove as well as double the failure rate of the glove (36). Currently, there are only dozens of sensors at most. If one cannot obtain a real sense of endoscopy feedback, then the endoscopy training provided by the Metaverse effect will be greatly limited (37).

### Remote endoscopic surgery in metaverse-endoscopic surgery across space

The advancement of remote endoscopic surgery could be considerably aided by the advent of Metaverse. Imagine a digestive expert using a VR helmet and a touch glove to access the Metaverse and become a virtual doctor, doing an endoscopy or even surgery on patients who are also represented in the Metaverse. The actual surgery process is performed by an AI robot in the real world. For more than two decades, remote surgery has been available. The first transatlantic cholecystectomy took place in 2001. Surgeons in New York City successfully removed a gall bladder from a 68-year-old woman in Strasbourg, France, using a remote-controlled robot and a high-speed cable video link. The time between when the surgeon performs an action and when it displays on the video screen is only 155 ms, thanks to the high speed of optical fiber propagation (38). As shown in this report, data transmission speed and surgical equipment are two critical factors in the success of remote endoscopic surgery (39).

### The impact of 5th generation mobile communication technology (5G)

The speed of remote surgery delay impacts the incidence of complications, and specialists discovered that many times <300 ms of operation time delay is required after the experiment (40). While relying on optical fiber can reduce operating delay, the development of the Metaverse will require a wireless telecommunications system and stability, speed, and current. This criterion has been mostly addressed by the introduction of 5G technology. 5G technology has been widely applied in the field of telemedicine (41). Since 2020, several remote laparoscopic experimental procedures utilizing the 5G wireless network have been performed successfully with an average delay of only 264 ms, with no surgical complications or prolonged operation time (42). An increasing number of 5G remote surgeries have recently been declared to be effective, including remote vocal cord surgery, remote spinal surgery, and even laser surgery for diabetic retinopathy patients (43–45). The Metaverse will be able to cover more locations in the future, including areas where the internet is difficult to lay out, such as the Arctic, thanks to the advancement of 5G technology.

### Long-distance endoscopic robot

A solid wireless communication foundation is also needed. Advanced surgical robots are ultimately responsible for the smooth execution of meta-cosmic endoscopic surgery (46). Remote surgery has become more common since the development of the Da Vinci surgical robot, while remote digestive endoscopy is still in the exploratory stage due to the lack of an advanced endoscopy robot. Remote digestive endoscopy is more commonly used for remote expert guidance, remote diagnosis, and other purposes (47). YUNSRobot, a new robot-assisted device, began performing upper gastrointestinal endoscopic model studies in 2020. Remote endoscopy using the YUNSRobot robot-assisted technology takes longer than on-site physician operations but still fulfills upper gastrointestinal endoscopy criteria. It is worth noting that the remote gastrointestinal endoscopy group's average surgical delay was 572.1 ± 48.5 ms in this study, which is still outside the safe limit for remote surgical delay time (48). To successfully implement remote endoscopic surgery in the meta-universe in the future, we must achieve extremely low network latency and have high-precision endoscopic robots. Because endoscopic surgery, such as EMR and ESD, has a very high risk of bleeding, massive bleeding in just a few seconds will make the operator completely lose the field of vision the focus and pose a great risk to the patient's life (49). Therefore, extremely low network delay and reliable endoscopic robots are very important. Currently, there is a low coverage and instability of 5G signals (50) or the backwardness of remote endoscopic robot technology (51). It is an important obstacle to implementing remote endoscopic surgery in metauniverses in the future.

## Discussion

The Metaverse combines virtual reality (VR), artificial intelligence (AI), blockchain, cloud computing, and a slew of other cutting-edge human technology to represent modern civilization's wisdom. At present, the Metaverse is making great strides toward us, and I believe that in the near future, the Metaverse will be widely used in all aspects of gastroenterology. The easiest thing to achieve is to use the Metaverse as a platform to participate in distance learning, which has already been realized at present. Referring to the Metaverse Classroom of Seoul National University, 200 well-known Asian cardiothoracic surgery experts gathered in a small Metaverse through VR equipment all over Asia. In the classroom, everyone turns into a virtual character of their choice, observes an operation together, exchanges surgical experience with each other, and participates in bachelor's lectures. Although this Metaverse is small, it is of great significance. I believe that in the near future, we will be able to access the Metaverse through various virtual devices, participate in world-class academic conferences, and observe endoscopic surgery in any corner of the world. Second, Metaverse is most valuable for gastroenterology in providing a virtual training platform for young endoscopists. In the future, we will be able to enter the Metaverse through VR simulators and tactile gloves, complete various difficult endoscopic treatment operations, and accumulate massive endoscopic experience. Of course, the current endoscopic VR simulators can still only provide the most basic models, which limits the improvement of the endoscopic skills of advanced endoscopists, and currently there are few patient models for endoscopic treatment. Similarly, with regard to tactile gloves, owing to the limitation of cost and the number of sensors, it is difficult for people to obtain realistic tactile sensations from the metaverse at this stage, which also greatly limits the training effect of simulated endoscopic operations in the metaverse. Finally, the most difficult but most meaningful, remote endoscopic surgery based on the Metaverse is bound to come. In the future, people can go through the Metaverse to receive examinations or treatments from endoscopists around the world. Of course, due to the low coverage rate of the current wireless communication infrastructure, the inability to achieve stable low latency, and the lack of remote endoscopic surgical robots, there is still much resistance to the real implementation of remote endoscopic surgery. The development of the Metaverse will also greatly benefit the remote medical treatment of patients, changing the existing medical treatment mode. In the future, registration, medical consultation, examination or remote endoscopic treatment can all be completed in the Metaverse. Although the current metaverse is in its infancy, we can still speculate on the important role of the metaverse in the field of gastroenterology in the future through the extensive application of the current metaverse technology in the medical field. I believe that the Metaverse is the dawn of a new era for gastroenterology, which will definitely change the study and work mode of gastroenterologists.

## Author contributions

CZ and SZ were involved in the conception of the study and critically revised the manuscript. SF, CZ, and RH were involved in writing the article. All authors read and approved the final manuscript.

## Funding

This work was supported by the National Natural Science Foundation of China (82074214). Funding was also provided by the Research Fund Project of Zhejiang Chinese Medical University (2019ZY02).

## Conflict of interest

The authors declare that the research was conducted in the absence of any commercial or financial relationships that could be construed as a potential conflict of interest.

## Publisher's note

All claims expressed in this article are solely those of the authors and do not necessarily represent those of their affiliated organizations, or those of the publisher, the editors and the reviewers. Any product that may be evaluated in this article, or claim that may be made by its manufacturer, is not guaranteed or endorsed by the publisher.

## References

[1] Wang D Yan XY. Research on metaverse: concept, development and standard system. 2021 In: 2nd International Conference on Electronics, Communications and Information Technology (CECIT), Sanya. (2021). p. 983–91.

[2] Hui P. Keynote Speaker: The hitchhiker's guide to the metaverse. In: 2022 IEEE Conference on Virtual Reality and 3D User Interfaces Abstracts and Workshops (VRW), Christchurch. (2022). p. 203.

[3] Kye B Han N Kim E Park Y Jo S. Educational applications of metaverse: possibilities and limitations. J Educ Eval Health Prof. (2021) 18:32. 10.3352/jeehp.2021.18.32 34897242 PMC8737403

[4] Xi N Chen J Gama F Riar M Hamari J. The challenges of entering the metaverse: An experiment on the effect of extended reality on workload. Inf Syst Front. (2022) 102:1–22. 10.1007/s10796-022-10244-x 35194390 PMC8852991

[5] Xie Y Zhang J Wang H Liu P Liu S Huo T. Applications of blockchain in the medical field: narrative review. J Med Internet Res. (2021) 23:e28613. 10.2196/28613 34533470 PMC8555946

[6] Gao F Thiebes S Sunyaev A. Rethinking the meaning of cloud computing for health care: a taxonomic perspective and future research directions. J Med Internet Res. (2018) 20:e10041. 10.2196/10041 29997108 PMC6060303

[7] Kamel Boulos MN Zhang P. Digital twins: from personalised medicine to precision public health. J Pers Med. (2021) 11:745. 10.3390/jpm11080745 34442389 PMC8401029

[8] Qureshi HN Manalastas M Ijaz A Imran A Liu Y Al Kalaa MO. Communication requirements in 5g-enabled healthcare applications: review and considerations. Healthcare. (2022) 10:293. 10.3390/healthcare10020293 35206907 PMC8872156

[9] Larentzakis A Lygeros N. Artificial intelligence (AI) in medicine as a strategic valuable tool. Pan Afr Med J. (2021) 38:184. 10.11604/pamj.2021.38.184.28197 33995790 PMC8106796

[10] Waschke KA Coyle W. Advances and challenges in endoscopic training. Gastroenterology. (2018) 154:1985–92. 10.1053/j.gastro.2017.11.293 29454788

[11] Koo H. Training in lung cancer surgery through the metaverse, including extended reality, in the smart operating room of Seoul National University Bundang Hospital, Korea. J Educ Eval Health Prof. (2021) 18:33. 10.3352/jeehp.2021.18.33 34965648 PMC8810683

[12] Skibba R. Virtual reality comes of age. Nature. (2018) 553:402–3. 10.1038/d41586-018-00894-w 32094793

[13] Ferlitsch A Glauninger P Gupper A Schillinger M Haefner M Gangl A. Evaluation of a virtual endoscopy simulator for training in gastrointestinal endoscopy. Endoscopy. (2002) 34:698–702. 10.1055/s-2002-33456 12195326

[14] Ahlberg G Hultcrantz R Jaramillo E Lindblom A Arvidsson D. Virtual reality colonoscopy simulation: a compulsory practice for the future colonoscopist? Endoscopy. (2005) 37:1198–204. 10.1055/s-2005-921049 16329017

[15] Koch AD Haringsma J Schoon EJ de Man RA Kuipers EJ. A second-generation virtual reality simulator for colonoscopy: validation and initial experience. Endoscopy. (2008) 40:735–8. 10.1055/s-2008-1077508 18698536

[16] Koch AD Ekkelenkamp VE Haringsma J Schoon EJ de Man RA Kuipers EJ. Simulated colonoscopy training leads to improved performance during patient-based assessment. Gastrointest Endosc. (2015) 81:630–6. 10.1016/j.gie.2014.09.014 25475901

[17] Blackburn SC Griffin SJ. Role of simulation in training the next generation of endoscopists. World J Gastrointest Endosc. (2014) 6:234–9. 10.4253/wjge.v6.i6.234 24932375 PMC4055992

[18] Kruglikova I Grantcharov TP Drewes AM Funch-Jensen P. The impact of constructive feedback on training in gastrointestinal endoscopy using high-fidelity Virtual-Reality simulation: a randomised controlled trial. Gut. (2010) 59:181–5. 10.1136/gut.2009.191825 19828469

[19] Harpham-Lockyer L Laskaratos FM Berlingieri P Epstein O. Role of virtual reality simulation in endoscopy training. World J Gastrointest Endosc. (2015) 7:1287–94. 10.4253/wjge.v7.i18.1287 26675895 PMC4673391

[20] Hashimoto DA Petrusa E Phitayakorn R Valle C Casey B Gee D. A proficiency-based virtual reality endoscopy curriculum improves performance on the fundamentals of endoscopic surgery examination. Surg Endosc. (2018) 32:1397–404. 10.1007/s00464-017-5821-5 28812161 PMC5809190

[21] Neumann M Mayer G Ell C Felzmann T Reingruber B Horbach T. The Erlangen Endo-Trainer: life-like simulation for diagnostic and interventional endoscopic retrograde cholangiography. Endoscopy. (2000) 32:906–10. 10.1055/s-2000-8090 11085482

[22] Grover SC Garg A Scaffidi MA Yu JJ Plener IS Yong E. Impact of a simulation training curriculum on technical and nontechnical skills in colonoscopy: a randomized trial. Gastrointest Endosc. (2015) 82:1072–9. 10.1016/j.gie.2015.04.008 26007221

[23] Walsh CM Scaffidi MA Khan R Arora A Gimpaya N Lin P. Non-technical skills curriculum incorporating simulation-based training improves performance in colonoscopy among novice endoscopists: Randomized controlled trial. Dig Endosc. (2020) 32:940–8. 10.1111/den.13623 31912560

[24] Piskorz MM Wonaga A Bortot L Linares ME Araya V Olmos JI. Impact of a virtual endoscopy training curriculum in novice endoscopists: first experience in Argentina. Digest Dis Sci. (2020) 65:3072–8. 10.1007/s10620-020-06532-8 32909122

[25] Khan R Plahouras J Johnston BC Scaffidi MA Grover SC Walsh CM. Virtual reality simulation training in endoscopy: a Cochrane review and meta-analysis. Endoscopy. (2019) 51:653–64. 10.1055/a-0894-4400 31071757

[26] Ward ST Hancox A Mohammed MA Ismail T Griffiths EA Valori R. The learning curve to achieve satisfactory completion rates in upper GI endoscopy: an analysis of a national training database. Gut. (2017) 66:1022–33. 10.1136/gutjnl-2015-310443 26976733

[27] Riva G Wiederhold BK. What the metaverse is (really) and why we need to know about it. Cyberpsychol Behav Soc Netw. (2022) 25:355–9. 10.1089/cyber.2022.0124 35696299

[28] Ahmed NM. Lataifeh visual pseudo haptics for a dynamic squeeze / grab gesture in immersive virtual reality 2021 *In: IEEE 2nd International Conference on Human-Machine Systems (ICHMS)*, Magdeburg. (2021). p. 1–4.

[29] Sun Z Zhu M Chen Z. Haptic-feedback ring enabled human-machine interface (HMI) aiming at immersive virtual reality experience. In: 21st International Conference on Solid-State Sensors, Actuators and Microsystems (Transducers), Orlando, FL. (2021). p. 333–6. 10.1109/Transducers50396.2021.9495698

[30] Ku J Mraz R Baker N Zakzanis KK Lee JH Kim IY. A data glove with tactile feedback for FMRI of virtual reality experiments. Cyberpsychol Behav. (2003) 6:497–508. 10.1089/109493103769710523 14583125

[31] Kar E Bose N Dutta B Mukherjee N Mukherjee S. Ultraviolet- and microwave-protecting, self-cleaning e-skin for efficient energy harvesting and tactile mechanosensing. ACS Appl Mater Inter. (2019) 11:17501–12. 10.1021/acsami.9b06452 31007019

[32] Sundaram S Kellnhofer P Li Y Zhu JY Torralba A Matusik W. Learning the signatures of the human grasp using a scalable tactile glove. Nature. (2019) 569:698–702. 10.1038/s41586-019-1234-z 31142856

[33] Sim D Baek Y Cho M Park S Sagar ASMS Kim HS. Low-latency haptic open glove for immersive virtual reality interaction. Sensors. (2021) 21:3682. 10.3390/s21113682 34070608 PMC8198336

[34] Junior JCVS Torquato MF Noronha DH Silva SN Fernandes MAC. Proposal of the tactile glove device. Sensors. (2019) 19:5029. 10.3390/s19225029 31752187 PMC6891499

[35] D'Abbraccio J Massari L Prasanna S Baldini L Sorgini F Airò Farulla G. Haptic glove and platform with gestural control for neuromorphic tactile sensory feedback in medical telepresence. Sensors. (2019) 19:641. 10.3390/s19030641 30717482 PMC6386988

[36] Caeiro-Rodríguez M Otero-González I Mikic-Fonte FA Llamas-Nistal M. A Systematic review of commercial smart gloves: current status and applications. Sensors. (2021) 21:2667. 10.3390/s21082667 33920101 PMC8070066

[37] Mylon P Lewis R Carré MJ Martin N Brown S. A study of clinicians' views on medical gloves and their effect on manual performance. Am J Infect Control. (2014) 42:48–54. 10.1016/j.ajic.2013.07.009 24268835

[38] Larkin M. Transatlantic, robot-assisted telesurgery deemed a success. Lancet. (2001) 358:1074. 10.1016/S0140-6736(01)06240-7 11589948

[39] Stanberry B. Telemedicine: barriers and opportunities in the 21st century. J Intern Med. (2000) 247:615–28. 10.1046/j.1365-2796.2000.00699.x 10886483

[40] Barba P Stramiello J Funk EK Richter F Yip MC Orosco RK. Remote telesurgery in humans: a systematic review. Surg Endosc. (2022) 36:2771-7. 10.1007/s00464-022-09074-4 35246740 PMC9923406

[41] Georgiou KE Georgiou E Satava RM. 5G use in healthcare: the future is present. JSLS. (2021) 25:e2021.00064. 10.4293/JSLS.2021.00064 35087266 PMC8764898

[42] Zheng J Wang Y Zhang J Guo W Yang X Luo L. 5G ultra-remote robot-assisted laparoscopic surgery in China. Surg Endosc. (2020) 34:5172–80. 10.1007/s00464-020-07823-x 32700149

[43] Acemoglu A Peretti G Trimarchi M Hysenbelli J Krieglstein J Geraldes A. Operating from a distance: robotic vocal cord 5G telesurgery on a cadaver. Ann Intern Med. (2020) 173:940–1. 10.7326/M20-0418 32658568

[44] Tian W Fan M Zeng C Liu Y He D Zhang Q. Telerobotic spinal surgery based on 5G network: the first 12 cases. Neurospine. (2020) 17:114–20. 10.14245/ns.1938454.227 32252160 PMC7136105

[45] Chen H Pan X Yang J Fan J Qin M Sun H. Application of 5G technology to conduct real-time teleretinal laser photocoagulation for the treatment of diabetic retinopathy. JAMA Ophthalmol. (2021) 139:975–82. 10.1001/jamaophthalmol.2021.2312 34236391 PMC8444028

[46] Lee N. Robotic surgery: where are we now? Lancet. (2014) 384:1417. 10.1016/S0140-6736(14)61851-1 25390313

[47] Costantino A Bortoluzzi F Giuffrè M Vassallo R Montalbano LM Monica F. Correct use of telemedicine in gastroenterology, hepatology, and endoscopy during and after the COVID-19 pandemic: Recommendations from the Italian association of hospital gastroenterologists and endoscopists (AIGO). Dig Liver Dis. (2021) 53:1221–7. 10.1016/j.dld.2021.06.032 34312103

[48] Yan B Liu H Yang YS Yang YM Peng LH Pan F. [The robot-assisted system YunSRobot for soft endoscopy: a trial of remote manipulation on simulation models]. Zhonghua nei ke za zhi. (2018) 57:901–6. 10.3760/cma.j.issn.0578-1426.2018.12.005 30486558

[49] Lau JYW Yu Y Tang RSY Chan HCH Yip HC Chan SM. Timing of endoscopy for acute upper gastrointestinal bleeding. N Engl J Med. (2020) 382:1299–308. 10.1056/NEJMoa1912484 32242355

[50] Ahamed MM Faruque S. 5G network coverage planning and analysis of the deployment challenges. Sensors. (2021) 21:6608. 10.3390/s21196608 34640928 PMC8512478

[51] Kume K Sakai N Ueda T. Development of a novel gastrointestinal endoscopic robot enabling complete remote control of all operations: endoscopic therapeutic robot system (ETRS). Gastroent Res Pract. (2019) 2019:6909547. 10.1155/2019/6909547 31781197 PMC6875422

